# Modifiable factors associated with Huntington's disease progression in presymptomatic participants

**DOI:** 10.1002/acn3.52120

**Published:** 2024-06-10

**Authors:** Andres Gil‐Salcedo, Renaud Massart, Laurent Cleret de Langavant, Anne‐Catherine Bachoud‐Levi

**Affiliations:** ^1^ Département d'Études Cognitives, École Normale Supérieure PSL University Paris 75005 France; ^2^ Faculté de Médecine Université Paris‐Est Créteil Créteil 94000 France; ^3^ Inserm U955, Institut Mondor de Recherche Biomédicale, Équipe NeuroPsychologie Interventionnelle Créteil 94000 France; ^4^ NeurATRIS, Mondor Node Créteil France; ^5^ APHP, Hôpital Henri Mondor, service de neurologie, centre national de référence maladie de Huntington Créteil 94000 France

## Abstract

**Objective:**

Huntington's disease (HD) is a neurodegenerative disorder characterized by progressive motor, cognitive, and psychiatric symptoms. Our aim here was to identify factors that can be modified to slow disease progression even before the first symptoms appear.

**Methods:**

We included 2636 presymptomatic individuals (comparison with family controls) drawn from the prospective observational cohort Enroll‐HD, with more than 35 CAG repeats and at least two assessments of disease progression measured with the composite Huntington's disease rating Scale (cUHDRS). The association between sociodemographic factors, health behaviors, health history, and cUHDRS trajectory was assessed with a mixed‐effects random forest using partial dependence plots and Shapley additive explanation method.

**Results:**

Participants were followed by an average of 3.4 (SD = 1.97) years. We confirmed the negative impact of age and a high number of CAG repeats. We found that a high level of education, a body mass index (BMI) <23 kg/m^2^ before the age of 40 and >23 kg/m^2^ thereafter, alcohol consumption of <15 units per week, current coffee consumption and no smoking were linked to slow disease progression, as did no previous exposure to antidepressants or anxiolytic, no psychiatric history or comorbidities, and being female. Other comorbidities or marital status showed no major association with HD evolution.

**Interpretation:**

Reducing modifiable risk factors for HD is one way to support the presymptomatic population. A high level of education, low‐to‐moderate alcohol consumption, no smoking, and BMI control are likely to slow disease progression in this population.

## Introduction

Huntington's disease (HD) combines motor, cognitive, and psychiatric symptoms, that progressively disable patients, with a median survival from motor onset of 18 years.^1^ HD is autosomal dominant and is caused by an expansion of cytosine‐adenine‐guanine (CAG) triplets in the Huntingtin gene^2^ (Htt). An increasing number of repetitions increases the risk of developing the disease, reduces the age at onset, and predicts a higher rate of decline. Age^2^, ^3^, ^4^, ^5^, ^6^ also associated robustly with progressive clinical deterioration.

Despite considerable research into disease‐modifying drugs, only environmental factors have been shown to impact disease progression.^6^, ^7^, ^8^ Moreover, disease trajectories differ between individuals with similar genotypic profiles and age at onset,^9^, ^10^ suggesting an influence of sociodemographic factors,^11^, ^12^, ^13^, ^14^, ^15^ health behaviors,^11^, ^16^, ^17^, ^18^, ^19^ or health history^11^, ^20^, ^21^, ^22^ on which intervention might have a positive impact. Identifying modifiable factors on which to act as early as possible is important to ensure the maximum effect of interventions and preserve a good quality of life for as long as possible. For instance, a high educational level is associated with an early diagnosis^12^ but also slower disease progression.^13^ Health behaviors such as high alcohol consumption,^16^ tobacco use,^18^ coffee consumption,^23^ and low body mass index (BMI)^19^ are associated with more rapid disease progression as are musculoskeletal,^24^ cardiovascular,^25^, ^26^ neurological,^27^ or psychiatric comorbidities,^20^ and a history of depression and anxiety.^21^ However, these associations remain unclear, and the evidence contrasted.

Previous analyses of factors associated with HD progression, despite the consistency of their results, faced some challenges. First, reverse causality, that is, when behaviors (e.g., smoking or drinking alcohol) or sociodemographic changes (e.g., work) are interpreted as causes of the disease when they are its consequence, or vice versa, as has already been observed in other conditions,^28^, ^29^ therefore studying modifiable factors before the disease manifest itself might address the reverse causality effect. Second, interactions between multiple factors are difficult to account for during disease progression,^16^ particularly because of the already described impact of the interaction between age and CAG repeat number. Third, factors such as BMI, alcohol consumption, or smoking have shown differential effects on function, mortality, or morbidity by age range, gender, or educational level in other populations,^30^, ^31^, ^32^, ^33^ but in HD these interactions remain unknown. One approach to account for these interactions is the use of bagging machine learning (ML) models that together with ML explanation methods such as SHapley Additive exPlanations (SHAP) allow estimating the contribution of factors on disease progression and their interactions without assumptions about their asociations.^34^ Thus, we aimed to identify, as early as the presymptomatic phase, the modifiable factors likely to influence the progression of HD, while identifying the interactions between these factors using an explained machine learning approach.

## Subjects/Materials and Methods

### Study population

Participants were included in the Enroll‐HD observational, prospective, international, multicenter study (NCT01574053). Ethical approval of the study was obtained, and all participants signed an informed consent. At enrolment, each participant was DNA genotyped to measure CAG repeats.^35^ The Enroll‐HD 2020 database is composed of 21,116 participants divided into mutation carriers, people without the mutation but with phenocopies (genotype negative), and family controls (Fig. S1). In total, 16,120 participants were considered mutation carriers, as they had a CAG number > 35. We retained those with complete sociodemographic and clinical data (*N* = 15,223).

The cUHDRS was used to identify presymptomatic mutation carriers as it summarizes the clinical state of HD gene carriers with different levels of functional impairment^8^ and has good biological relevance.^36^ The cUHDRS combines functional (Total Functional Capacity (TFC)), motor (Total Motor Score (TMS)), and cognitive measurements (Symbol Digit Modalities Test (SDMT) and Stroop Word Reading (SWR)) extracted from annual assessments using the following formula:

$$\text{cUHDRS}=\begin{array}{l} \left(\mathrm{TFC}-10.4\right)/1.9–\left(\mathrm{TMS}–29.7\right)/14.9\\ +\;\left(\text{SDMT}–28.4\right)/11.3+\left(\mathrm{SWR}–66.1\right)/20.1+10. \end{array}$$

A lower cUHDRS indicates a more advanced disease.

A participant was considered presymptomatic when his/her cUHDRS at baseline was higher than the lower bound of the 95% confidence interval of prediction with a mixed model calibrated with cUHDRS of family controls (*n* = 2357, Fig. S1) adjusted for age, sex, education level, marital status, health behavior, and the number of comorbidities (Supplementary Methods).

### HD progression

HD progression was evaluated with changes in the cUHDRS score.^37^, ^38^

### Modifiable and non‐modifiable factors assessment

Sex (female vs. male), age at each assessment, and CAG repeats were included as non‐modifiable factors. Other sociodemographic factors at baseline included marital status (single [single, divorced, widowed, separated] vs. in a couple [married, partnership]), education level (international standard classification of education [ISCED‐1997], levels from 0 = early childhood education to 6 = doctoral or equivalent level), and residence (city vs. elsewhere). Health behaviors at baseline included alcohol consumption (self‐report of units of alcohol by week), tobacco consumption (yes vs. no), coffee consumption (no, currently and more than 3 cups per day), drug use (yes vs. no), and BMI (kg/m^2^). History of cardiovascular, metabolic, neurological, and psychiatric diseases, as well as other morbidities were included in the health history at baseline. A history of pharmacological treatment for anxiety and depression at baseline was also included.

### Statistical analysis

The population's characteristics were described based on the change in cUHDRS between the baseline and the last available assessment in each patient, with the population split into two groups (Low‐ΔcUHDRS vs. High‐ΔcUHDRS, see supplemental methods for details). Categorical variables were assessed using Pearson's chi‐squared test, and continuous variables using a *t*‐test, to compare baseline characteristics of the two groups.

Associations between modifiable and non‐modifiable factors and cUHDRS trajectories were identified using mixed‐effects random forest (MERF) calibration.^39^ For model calibration, individuals were included as random effects at intercept and slope with age as the timescale. Age at interview, CAG repeats, sociodemographic factors, health behaviors, and health status variables were included as fixed effects. The explanation of the MERF model results and assessment of the interactions were carried out using the SHapley Additive exPlanation method (SHAP).^40^ Please refer to the supplementary methods for additional information on the descriptive, main, and sensitivity analyses.

## Results

**Figure 1:**
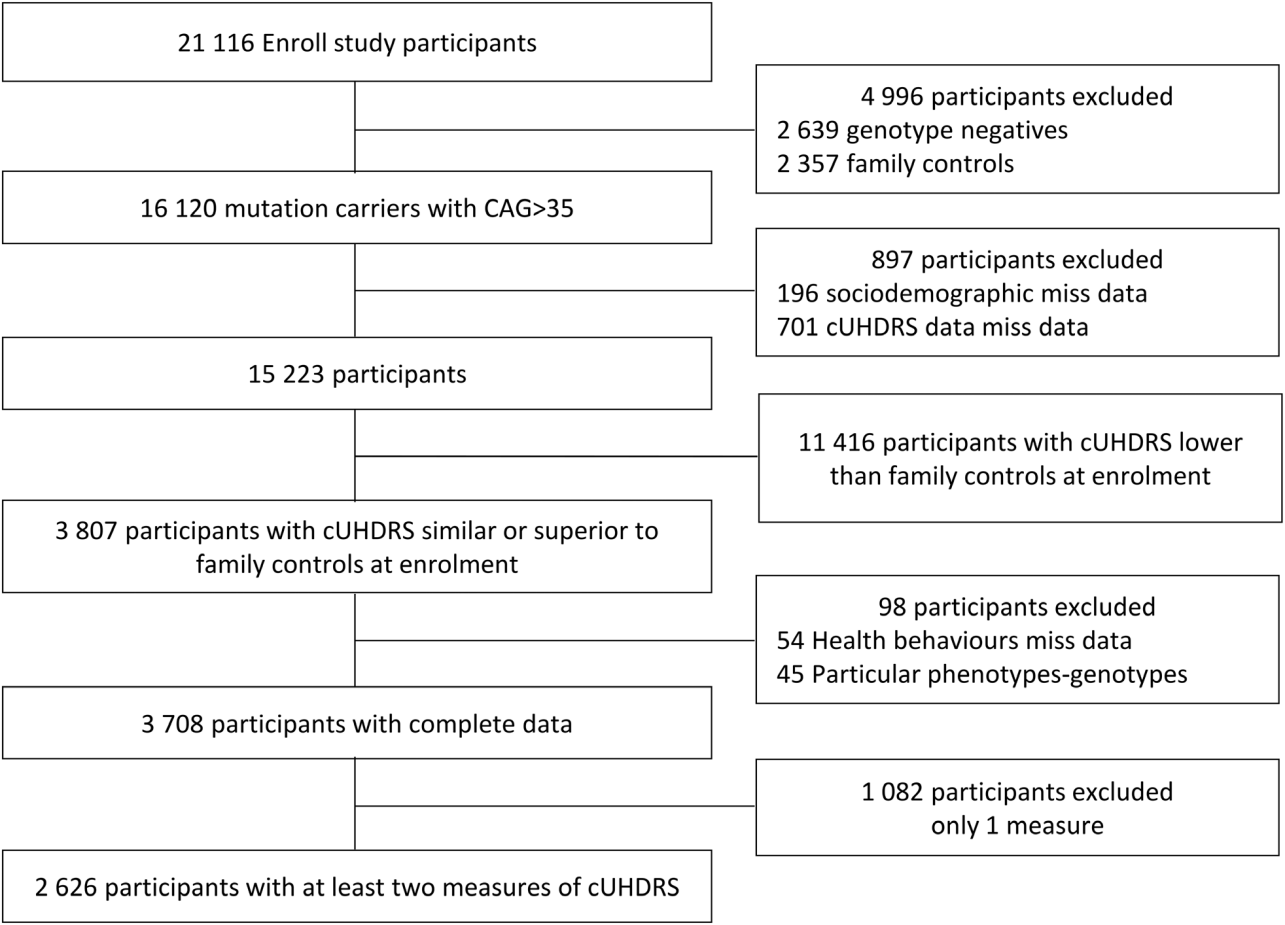
Analysis of population selection flowchart. The extended flowchart in Figure S1.

Of the 15,223 mutation carriers (>35 CAG repeats) with complete sociodemographic and cUHDRS data (Fig. 1, Table S1), 11,416 showed a lower cUHDRS at enrolment than controls and were therefore excluded. Of the 3807 remaining participants who had comparable or higher cUHDRS than controls at enrolment and henceforth called presymptomatic HD mutation carriers, 54 participants were excluded because of missing data on health behaviors and 45 because of off‐norm cUHDRS trajectories (very rapid and early decline of patients with a CAG number >55 or lack of decline despite advanced age). Finally, 1082 participants were excluded because of an absence of longitudinal follow‐up. A total of 2626 presymptomatic mutation carriers were included in this study (Fig. 1, Table S1).

The presymptomatic population (*N* = 2626) was composed of 57.5% women, with a mean age of 40.16 (SD = 12.6) and a mean follow‐up of 3.40 years (SD = 1.97, Table 1). The mean difference between the first and last cUHDRS measurements adjusted for follow‐up time was (ΔcUHDRS/time) = −0.15 (SD = 0.7). The slow decliners had a difference smaller than the absolute mean time‐adjusted (Low‐ΔcUHDRS = ΔcUHDRS/time < =|−0.15|), and the fast decliners, greater (High‐ΔcUHDRS = ΔcUHDRS/time > |−0.15|).

**Table 1:** Characteristic of groups of presymptomatic mutation carriers based on the total difference between the first and last measure of cUHDRS.^a^

Characteristics	Total (*N* = 2626)	Slow decliner	Faste decliners	*p*‐Value
		Low‐ΔcUHDRS (*N* = 1524)	High‐ΔcUHDRS (*N* = 1102)	
Sex				
Women	1510 (57.5%)	882 (58.7%)	628 (55.9%)	0.144
Man	1116 (42.5%)	620 (41.3%)	496 (44.1%)	
Age, mean (SD)	40.16 (12.60)	38.02 (11.81)	43.02 (13.05)	<0.001
CAG repeats, mean (SD)	41.97 (2.52)	41.78 (2.44)	42.22 (2.59)	<0.001
Education level, mean (SD)	3.92 (1.16)	3.98 (1.13)	3.83 (1.19)	0.002
Marital status				
Single	877 (33.4%)	531 (35.4%)	346 (30.8%)	0.014
In couple	1749 (66.6%)	971 (64.6%)	778 (69.2%)	
Residence in city				
Non	1429 (54.4%)	800 (53.3%)	629 (56.0%)	0.169
Yes	1197 (45.6%)	702 (46.7%)	495 (44.0%)	
Alcohol consumption (units), mean (SD)	3.90 (6.10)	3.55 (5.51)	4.37 (6.78)	<0.001
Tobacco consumption				
Non	2010 (76.5%)	1153 (76.8%)	857 (76.2%)	0.756
Yes	616 (23.5%)	349 (23.2%)	267 (23.8%)	
Coffee consumption				
Non	976 (37.2%)	556 (37.0%)	420 (37.4%)	0.012
Currently	969 (36.9%)	585 (38.9%)	384 (34.2%)	
More than three cups per day	681 (25.9%)	361 (24.0%)	320 (28.5%)	
Abused drugs				
Non	2243 (85.4%)	1295 (86.2%)	948 (84.3%)	0.178
Yes	383 (14.6%)	207 (13.8%)	176 (15.7%)	
BMI (kg/m^2^)	26.01 (5.36)	25.97 (5.37)	26.07 (5.35)	0.652
Comorbidities				
Cardiovascular	349 (13.3%)	182 (12.1%)	167 (14.9%)	0.041
Metabolic	331 (12.6%)	166 (11.1%)	165 (14.7%)	0.006
Neurologic	393 (15.0%)	201 (13.4%)	192 (17.1%)	0.009
Psychiatric	710 (27.0%)	349 (23.2%)	361 (32.1%)	<0.001
Others	1836 (69.9%)	1030 (68.6%)	806 (71.7%)	0.083
Pharmacological treatment for				
Anxiety	182 (6.9%)	81 (5.4%)	101 (9.0%)	<0.001
Depression	402 (15.3%)	180 (12.0%)	222 (19.8%)	<0.001
cUHDRS, mean (SD)	17.16 (1.38)	17.32 (1.16)	16.95 (1.60)	<0.001
TFC	12.88 (0.50)	12.92 (0.38)	12.82 (0.61)	<0.001
TMS	2.56 (4.14)	1.79 (2.99)	3.58 (5.12)	<0.001
SDMT	54.47 (9.78)	55.41 (8.99)	53.23 (10.63)	<0.001
Stroop	100.78 (13.94)	100.79 (13.08)	100.77 (15.02)	0.966
Follow‐up, mean (SD)	3.40 (1.97)	3.38 (1.92)	3.42 (2.04)	0.574

SDMT, Symbol Digit Modalities Test; TFC, total functional capacity; TMS, total motor score.

^a^ Low‐ΔcUHDRS: presymptomatic mutation carriers with a ΔcUHDRS/time below the time‐adjusted absolute mean; High‐ΔcUHDRS: presymptomatic mutation carriers with a ΔcUHDRS/time during follow‐up above the time‐adjusted absolute mean; mean time‐adjusted difference in cUHDRS score = −0.15 (SD = 0.7).

Fast decliners were more likely older, with a higher number of CAG repeats, lower level of education, often in a couple, and consumed more units of alcohol per week (*p* < 0.014 for all) compared to slow decliners. They also had a more frequent history of cardiovascular, metabolic, neurological, and psychiatric morbidities, as well as a history of pharmacological treatment for depression and anxiety (*p* < 0.041 for all) compared so slow decliners.

The MERF fully adjusted model showed a mean absolute error (MAE) of 0.24 points of cUHDRS and a mean square error (MSE) of 0.10 in the training sample (Fig. S2, Panel A). In the validation sample, the MAE and MSE were 0.74 and 1.06, respectively. The per‐subject betas associated with the random effect showed a normal distribution (Fig. S2, Panel B).

**Figure 2:**
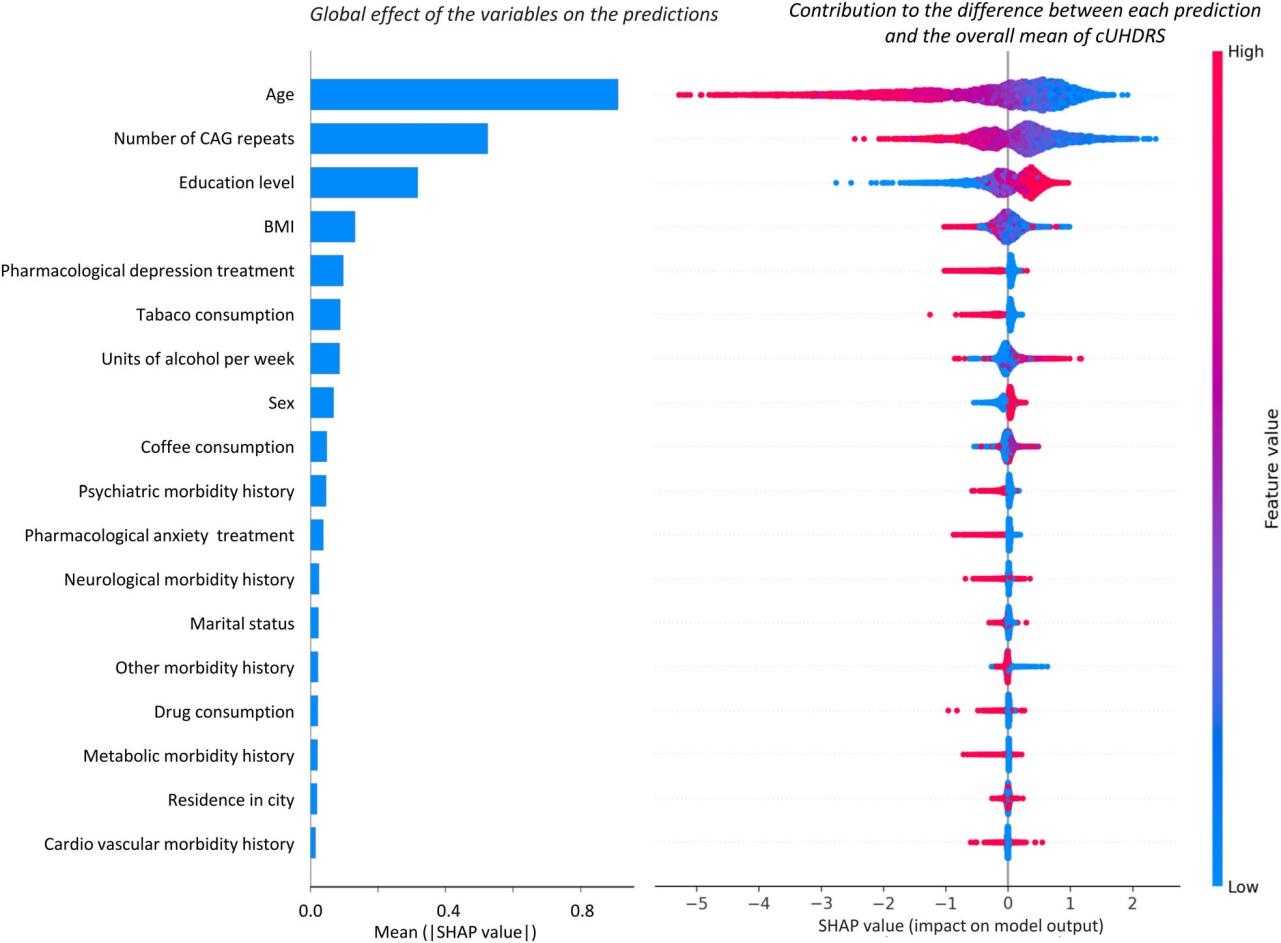
Summary plot of the SHAP values for each prediction organized by level of relevance of each variable. The left‐hand panel shows the relative importance of variables, expressed as mean (|shap| values) in the fully adjusted mixed‐effects random forest model. The right‐hand panel shows the SHAP values for each of the individuals, represented by single dots, per variable and as a function of variable values. The color of each dot represents the value of each variable. The redder the dot, the higher the value of the variable and the bluer, the lower the value. Variable ranges are age [18–80], number of CAG repeats [36–55], education level [0–6 ISCED levels], BMI [18–36], pharmacological depression and anxiety treatment [0 = No‐1 = Yes], units of alcohol per week [0–30], tobacco and drugs consumption [0 = No‐1 = Yes], coffee consumption (0 = non, 1 = currently, 2 = more 3 cups per day), sex [0 = man‐1 = woman], all comorbidities history [0 = No‐1 = Yes], marital status [0 = single, 1 = in couple], and residence in a city [0 = No‐1 = Yes]. Adjusted linear mixed model with the first 9 variables is described in detail in Table S2 (*p*‐value <0.035 for all variables except for history of psychiatric morbidity, and interaction between educational level and age).

The SHAP method predicted an overall adjusted mean cUHDRS for presymptomatic HD mutation carriers of 17.02. The right‐hand panel of Figure 2 shows the SHAP values for each of the individuals, represented by single dots, per variable and as a function of variable values (see example of interpretation in supplemental results and Fig. S3). Age and CAG number repeats were the variables that contributed most to the prediction, with a mean(|shap|) of 0.91 and 0.53 respectively. Educational level, BMI, and history of antidepressant treatment contributed with a mean(|shap|) of 0.32, 0.13, and 0.10, respectively. Tobacco consumption, units of alcohol per week, sex and coffee consumption contributed with a mean(|shap|) of 0.09, 0.09, 0.07, and 0.05, respectively. The sum of the other eight variables was close to a mean(|shap|) of 0.19 (all mean(|shap|) ≤ 0.04).

**Figure 3:**
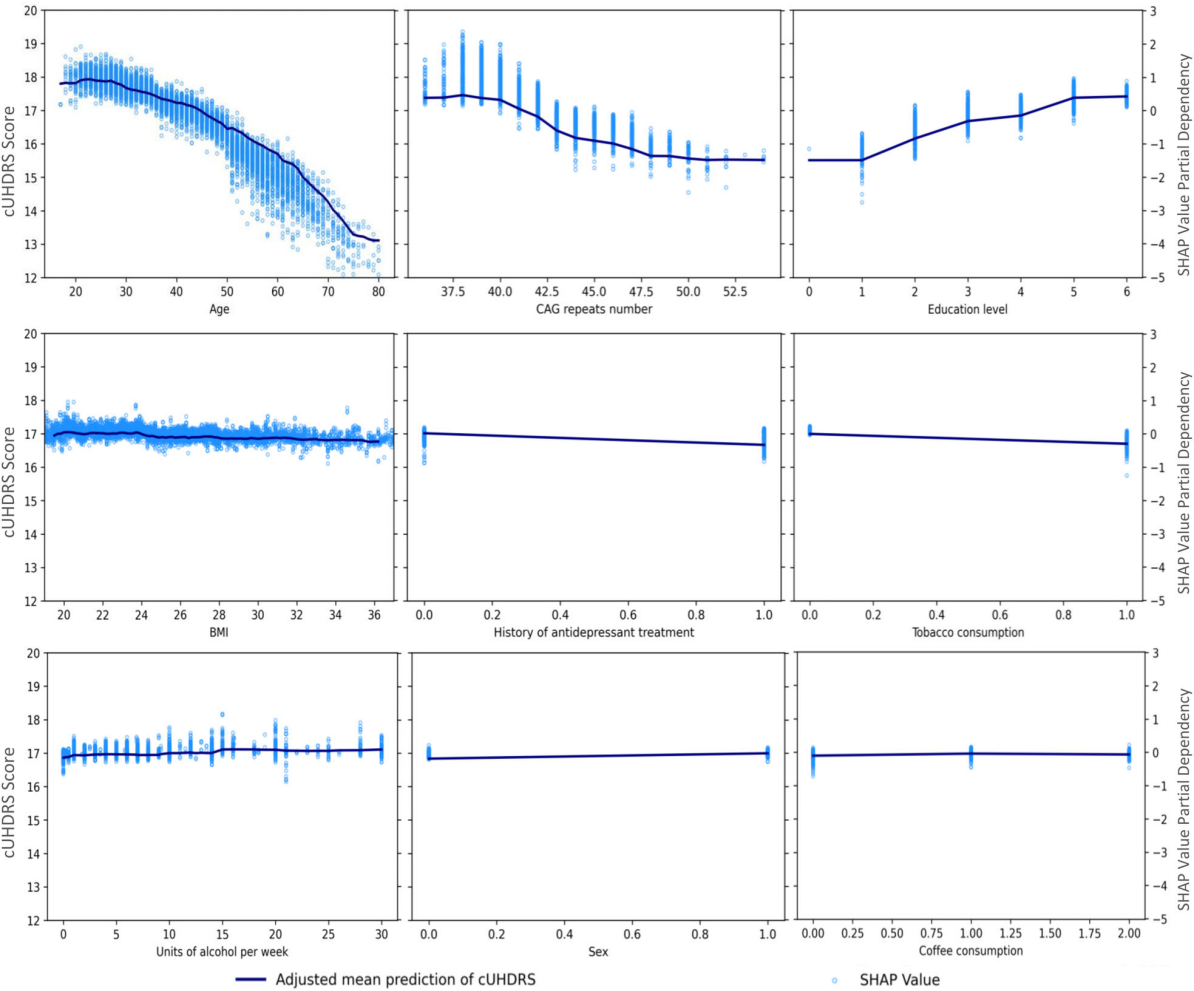
Partial dependence and SHAP values of the most relevant variables for prediction and cUHDRS. Mean partial dependency from MERF fully adjusted model. SHAP values of the predictions of fully adjusted mixed‐effects random forest model. Ranges of feature value: age [18–80], number of CAG repeats [36–55], education level [0–6 ISCED levels], BMI [18–36], pharmacological depression and anxiety treatment [0 = No‐1 = Yes], units of alcohol per week [0–30], tobacco and drugs consumption [0 = No‐1 = Yes], coffee consumption (0 = non, 1 = currently, 2 = more 3 cups per day), sex [0 = man‐1 = women], all comorbidities history [0 = No‐1 = Yes], marital status [0 = single, 1 = in couple], and residence in a city [0 = No‐1 = Yes]. PD: partial dependency. Adjusted linear mixed model with the first 9 variables is described in detail in Table S2 (*p*‐value <0.035 for all variables except for history of psychiatric morbidity, and interaction between educational level and age).

Figure 3 shows the partial dependence plot of the change in cUHDRS when each variable changes but assumes other variables are constant (marginal effect) and the corresponding SHAP value for each prediction. The cUHDRS decreased by about 5.0 points between the ages of 25 and 80, with accelerated decline with older age. When it comes to the number of CAG repeats, the cUHDRS showed a decline of approximately 1.7 points from 38 to 47 CAG repeats. However, the decline is less significant and around 0.3 cUHDRS points from 47 to 55 CAG repeats.

**Figure 4:**
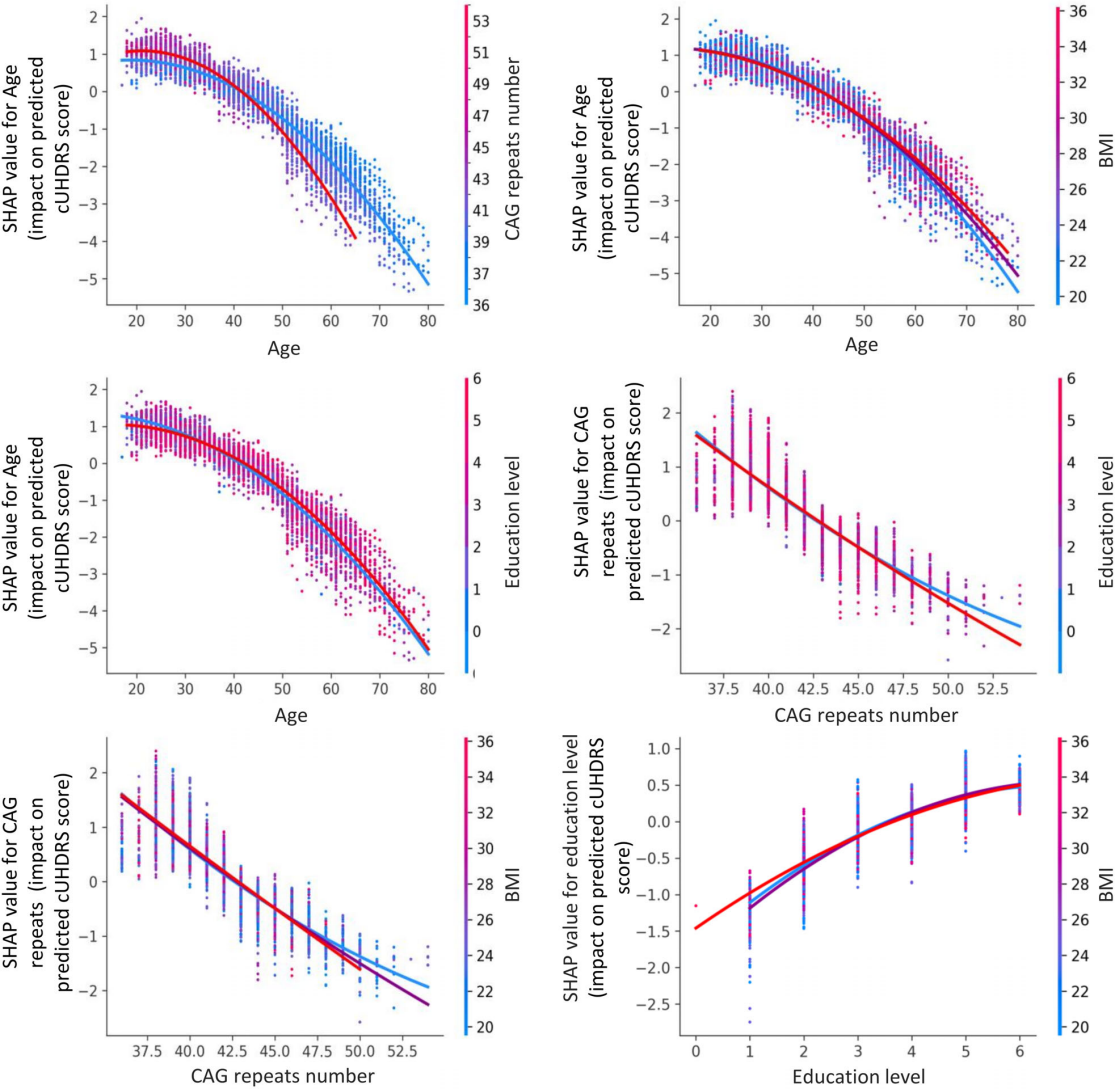
cUHDRS partial dependence plot with SHAP values of age and CAG repeats number with most relevant trajectories of the interacting variables in the mixed‐effects random forest model. SHAP values of the predictions of fully adjusted mixed‐effects random forest model. CAG repeat number blue<42≤red. Body index mass (BMI) blue = [12.5–23.1], purple = [23.2–27.0], and red= [27–50]. Education level blue<4≤red. SHAP interaction values in Figure S5.

All interactions between variables were examined and based on the mean (|shap|) of each interaction. The most important interactions are shown in Figure 4 (more details in Fig. S4). We confirmed that cUHDRS decreased more rapidly with age in presymptomatic mutation carriers with CAG repeats above 42 (median) than in those with CAG repeats below 42 (SHAP values of interaction: CAG‐repeats × age mean(|shap|) = 0.38) (Fig. S5). In terms of modifiable factors, an increase in education level from 1 to 5 is associated with a mean of 1.8‐point increase in cUHDRS (Fig. 3). However, this effect interacts with age, with education levels 4–6 considered protective between the ages 35–70, after which the effect diminishes (education‐level × age mean(|shap|) = 0.16, Figure 4). A BMI between 20 and 23 kg/m^2^ had a mean of cUHDRS close to 17.0, that is, 0.14 points higher on mean than presymptomatic mutation carriers with a BMI between 23 and 35 kg/m^2^ (Fig. 3).

When considering the effects of age on presymptomatic mutation carriers, those with a BMI below 23.1 kg/m^2^ (in the first tertile) had a better cUHDRS between the ages of 25 and 40 compared to the rest of the presymptomatic population. However, after this age range, presymptomatic mutation carriers with a BMI above 27 kg/m^2^ had a better cUHDRS with increasing age compared to those with a BMI between 23.2 and 27.0 kg/m^2^, and even more so compared to those with a BMI below 23.1 kg/m^2^ (BMI × mean age(|shap|) = 0.13).

Regarding alcohol consumption, the cUHDRS increased by around 0.25 points in those who consumed 16 units of alcohol per week compared with those who did not drink alcohol (Fig. 3). Beyond 16 units, less pronounced changes were observed in the cUHDRS, with a tendency to decrease or stabilize, probably due to the small number of participants. In addition, presymptomatic mutation carriers who had not used tobacco saw their cUHDRS increased by almost 0.30 points compared with tobacco users. Presymptomatic mutation carriers exposed to antidepressants showed a mean decrease of 0.34 points in cUHDRS compared with those not exposed (Fig. 3). Females showed a mean increase of 0.18 in the model compared with males. Participants who reported recurrent coffee consumption showed a mean increase of 0.06 in the model compared to those who did not consume coffee and 0.02 compared to those who consumed more than three cups per day. Finally, a history of psychiatric morbidity, marital status, city residence, drug use, exposure to anxiolytics, and history of other morbidities showed a contribution of <0.1 points in predicting the cUHDRS. Interactions between the number of CAG repetitions and education and BMI, as well as between education level and BMI, were suggested by the SHAP method with evidence of slight differences between groups.

Refer to supplementary Results for details on sensitivity analysis, Figures S6–S9, Tables S2 and S3.

## Discussion

We analyzed longitudinally 2626 mutation carriers who were presymptomatic at the time of enrolment in the Enroll‐HD cohort, to identify modifiable and non‐modifiable factors associated with progression of HD. We carefully matched the presymptomatic HD population with healthy controls using the cUHDRS a composite score assessing disease progression and a statistical model adjusted with factors known to have an impact on disease evolution. First, we confirmed the negative impact of higher number of CAG repeats and increasing age on the rate of HD progression. In contrast, being female had a protective effect on disease progression. Regarding modifiable factors, we showed that a high educational level was protective from age 35 onward. In contrast, BMI below 23 kg/m^2^ may be protective before the age of 40 but may increase the risk of more rapid disease progression after 40. In addition, moderate alcohol consumption (up to 15 units per week), abstinence from smoking, coffee current consumption, and lack of exposure to antidepressants were associated with slower progression of HD.

Our analysis confirmed the negative impact of CAG repeats and age on the rate of HD progression.^2^, ^3^, ^4^, ^5^, ^6^ On the other hand, we found that being a woman had a protective effect, in contrast to previous studies which showed slightly faster progression in women with HD.^22^ The association between sex differences and HD, whether in terms of prevalence, symptom severity or dynamics, remains to be clarified. Indeed, the literature suggests complex associations leading to results that are difficult to reconcile at first glance. No sex difference regarding the age of onset of the disease, generally defined as the onset of motor symptoms, has been reported.^15^, ^22^ However, studies have found that women present poorer functional and motor scores at the first visit^22^ and during HD evolution^15^ but a longer duration of illness.^41^

Regarding modifiable factors, we showed that a high educational level was protective from the age of 35. This effect may be attributed to a cognitive reserve,^12^, ^42^ which is influenced by education and reflects increased brain resilience. While education level is associated with socioeconomic status and its modification, participation in cognitively demanding or cognitive reinforcement activities can serve as a tool to promote cognitive reserve. People with a high level of education are generally diagnosed earlier than those with an average or low level of education,^12^ which can increase the likelihood of benefiting from cognitive reinforcement interventions or changes in environment and daily routines before presenting severe symptoms. Consequently, it cannot be excluded that the beneficial effect of a high level of education may be more attributable to the early detection of the disease than to the education itself.

We observed that smoking abstinence was associated with a slower decline in health status, in line with other studies showing an earlier age of onset in smoker.^18^ However, our findings contrast with those of other studies of HD participants, where tobacco use did not impact disease progression.^11^ This discrepancy in results may be due to differences in the populations analyzed and the methods employed. Our study focused on participants who began follow‐up without apparent symptoms of HD, whereas the study by Griffin et al. included individuals who had already exhibited symptoms. Their methodology sought to balance the samples with propensity scores according to each factor. A sensitivity analysis with propensity scores was conducted in our study also to minimize the potential bias of an observational study. The results were found to be consistent with the main findings (Table S3).

Surprisingly, we found that alcohol consumption had a protective effect of up to 15 units of alcohol per week. This result has been reported in other chronic conditions such as frailty,^43^ dementia,^44^ and certain cardiovascular diseases.^45^ Nevertheless, the association between alcohol abuse and behavioral problems, and depression present in HD,^46^ as well as the association of alcohol consumption with all causes of mortality must be highlighted.^31^ Furthermore, like others, our results are based on self‐reporting of alcohol units and do not examine the type of alcohol, which might limit the confidence in these results like in other studies. On the other hand, HD participants with excessive alcohol consumption and severe behavioral symptoms are often excluded a priori from observational studies.^47^ Our study includes around 5.3% of participants with alcohol consumption of over 15 units per week, which seems rather low. Consequently, our findings concerning the effects of alcohol consumption should be taken with caution.

We observed an interaction between BMI and age: after the age of 40, participants with a BMI >23 may be protected compared with those with a BMI <23, which confirms previous studies.^19^ However, our results showed a protective effect of a BMI below 23 in participants aged under 40, which had not been observed until now. This might be a consequence rather than a cause, weight loss is often a turning point in the progression of the disease and may mask the metabolic predictors of the disease.^19^, ^48^

Our study describes an association at the presymptomatic stage between antidepressant exposure and disease progression, in agreement with what is known at more advanced stages of the disease.^11^, ^49^ It was even suggested that depression may correspond to an early symptom of HD, not explained by concerns at being at risk, but reflecting early manifestation of neuronal dysfunction.^50^, ^51^ Our study underlines the importance of closer monitoring by mental health services of the HD population. Episodes of depression or psychiatric medical history may be indicators of an accelerated, if not early, evolution of HD. However, this latter hypothesis should be further studied to provide more evidence and discover its mechanisms. In our main results, coffee consumption showed a protective effect on the evolution of HD. However, when evaluating the cUHDRS components separately, the protective effect was observed in TFC and was less marked in TMS. In contrast, the effect of coffee consumption on SDMT showed a detrimental effect (Fig. S7). This deleterious effect was observed in a previous study where premanifest HD participants who consumed coffee showed lower cognitive performance compared to those who did not consume coffee.^23^ Nevertheless, the precise mechanism underlying this relationship remains unclear in premanifest HD, and further studies are needed to elucidate this relationship.

Our findings do not provide evidence of a strong association between a history of morbidities prior to HD symptoms and HD progression. However, a possible protective effect has been observed between immunomodulatory and antihypertensive drug treatments received in HD patients with comorbidities such as hypertension or sclerosis.^52^, ^53^ Other treatments, such as antidepressants, sildenafil, and selective serotonin reuptake inhibitors, have also been associated with better outcomes in HD^49^, ^54^, ^55^. However, our study was limited to evaluating the effect of antidepressant exposure, and the results were consistent with those of previous studies. Therefore, further studies should be conducted to specifically examine multimorbidity and the impact of multi‐medication on the evolution of HD and pre‐HD populations. On the other hand, this study evaluated the effect of non‐medical drug use, and we did not find a robust association with disease progression. However, we did not evaluate the specific effect of each substance. Previous studies have discussed the possible protective effect of cannabis on disease onset,^17^ but the results were not confirmed in subsequent studies.^11^ Additional research is required to assess the impact of drug exposure, such as cannabis, in the presymptomatic phase and its subsequent effect on HD.

Previous studies explored several factors in symptomatic HD participants. Thus, any protective or detrimental effect of a factor could be either the consequence or the cause of disease progression.^28^ Here we studied the impact of modifiable and non‐modifiable factors in a carefully selected presymptomatic population based on the comparison of cUHDRS between HD carrier mutation and controls, considering both sociodemographic variables, morbidities, and health behaviors. This approach may reduce the risk of reverse causality in epidemiological studies like ours, compared to methods such as the HD‐ISS, which showed their relevance in clinical research studies. Our analyses were based on a reliable and flexible modeling method; the machine learning random forest, to which a random effect per individual was added, allowing greater modeling accuracy with a low risk of overfitting.^34^ This method enabled us to assess the association of each factor without presuming the shape of the association and to consider all possible interactions likely to influence the prediction. In addition, the use of SHAP values to understand the association between risk factors and HD progression, and their interactions, enabled us to draw reliable conclusions. This is despite the complexity that can exist in interpreting machine learning models.

The results of our work must be interpreted in the light of its limitations. (1) The findings presented herein pertain to the selection of participants without apparent symptoms of HD at baseline. Consequently, the results should not be extrapolated to HD patients with manifestation of symptoms. (2) Despite a longer follow‐up than in prior studies, the follow‐up period was not long enough to study participants reaching a cUHDRS score of <8 points, which may have led to an underestimation of the impact of factors on HD progression because of the under‐representation of individuals at an advanced stage of the disease. (3) The number of participants with only one cUHDRS measure who were excluded represented approximately 29% of the study population. This population was similar to the study population in terms of sociodemographic variables and alcohol consumption but had fewer comorbidities despite higher tobacco and drug use than the study population (Table S1). (4) Data on certain health behaviors such as physical activity or diet were not available. However, a previous study did not observe an association between active lifestyle and age at disease onset.^56^ Nevertheless, the hypothesis arises as to whether a passive lifestyle may be a preclinical expression of HD. Future research should explore the role of diet and physical activity as potential modifiable factors.

## Conclusion

Education, low‐to‐moderate alcohol consumption, current coffee consumption, and nonsmoking may be protective factors against disease progression before onset. A BMI below 23, may be protector before the age of 40. In addition, mental health services may prove critical in the follow‐up of presymptomatic mutation carriers. Multi‐domain management of above risk factors could be an efficient prevention strategy to delay the onset and slow the progression of HD since the presymptomatic phase. Such approach might also be effective for other neurodegenerative diseases, in which prevention based on modifiable factors may compensate the lack of cure.

## Author Contributions

Conceptualization: AG, RM, and ACBL; Methodology: AG and RM; Validation: ACBL and LCL; Formal analysis: AG and RM; Data Curation: AG; Writing—original draft preparation: AG, RM, and LCL; Writing—review and editing: ACBL; Visualization: AG, RM, and ACBL; Supervision: ACBL.

## Funding Information

This work was supported by the French national infrastructure NeurATRIS (Investissement d'Avenir‐ANR‐11‐INBS‐0011) and the Fondation pour la Recherche Médicale, grant number SPF202309017513, to AGS.

## Conflict of Interest

The authors declare that the research was conducted in the absence of any commercial or financial relationships that could be construed as a potential conflict of interest.

## Supporting information


Data S1.

## Data Availability

Periodic data set available through the Enroll‐HD website (enroll‐hd.org/), privacy measures and data security are described in website.
