# Supplementary material for: Modifiable factors associated with Huntington's disease progression in presymptomatic participants
Source: Ann Clin Transl Neurol. 2024 Jun 10;11(7):1930–41. doi: 10.1002/acn3.52120 (PMC11251488; doi:10.1002/acn3.52120)
Supplement: Supplementary file 1 — Data S1. [file ACN3-11-1930-s001.docx]

Supplementary Methods

*Study population using cUHDRS*

Participants with CAG between 35 and 55 in a presymptomatic stage were selected based on their cUHDRS score at the first evaluation. Those who presented an adjusted cUHDRS similar to or above that of the control population were selected using the following process:

1. From the 21 116 Enroll-HD participants, 2357 control families were retained. The 2639 genotype-negative participants were excluded due to overexposure to an HD context (participants with parents with HD). 49 participants were excluded because of missing sociodemographic data, health behaviors, or morbidities.
2. With the 2308 participants, a linear mixed model was fitted with cUHDRS as the outcome and with age as the time scale so its quadratic form. A random effect was included at the intercept and the slope at the individual level. The model was adjusted sequentially for sex, education level, marital status, alcohol consumption, coffee consumption, tobacco consumption, BMI, and the number of comorbidities so that an interaction between age and education level.
3. Based on the previously adjusted model, the cUHDRS corresponding to each of the participants with a CAG of 36 to 55 was estimated for their characteristics (age, sex, level of education, health behavior, BMI and the number of comorbidities) using the fixed effect of the model. an adjusted mean cUHDRS and its 95% confidence interval were estimated using simulations of all model parameters to obtain 1000 predictions. Details in the *merTools::predictInterval* documentation (version0.6.1).
4. Participants with CAG>35 with an observed cUHDRS at first measure above the lower bound of the confidence interval estimated by the previous model were considered as presymptomatic HD and were included in the target population.

HD mutation carriers in the presymptomatic phase, as well as the general population, may have low motor, cognitive, or functional scores associated with chronic conditions other than HD (e.g., psychiatric, musculoskeletal, metabolic, or neurological conditions) or with unfavorable health behaviors (high alcohol consumption, smoking, or obesity). Therefore, we prioritized selecting participants by adjusting our main outcome model for sociodemographic factors, comorbidities, and health behaviors. The aim of this approach is not to exclude pre-symptomatic participants with low scores. Other selection methods, such as the HD-ISS classification (which selects participants with CAG≥40 based on their TMS, SDMT, educational level, independence scale and Total Functional Capacity), could potentially exclude carriers of mutations with low scores related to risks other than HD itself.

*Statistical analysis detailed*

The description of population characteristics and their association with change in cUHDRS over time established two groups of presymptomatic mutation carriers at baseline based on the rate of change in cUHDRS between the first and last assessment (ΔcUHDRS/Time between the two assessments). The first group consisted of presymptomatic whose rate of change in cUHDRS was lower than the time-adjusted total presymptomatic population mean (low-ΔcUHDRS) and the second group of those whose rate was higher (high-ΔcUHDRS). Comparison of each variable between the two groups provided an overview of the crude associations between cUHDRS change and population characteristics. Baseline characteristics between groups were assessed using Pearson's chi-2 test for categorical variables (sex, marital status, residence, smoking, drug use, comorbidities, and pharmacological treatments) and a t-test for continuous variables (age, CAG, education level, units of alcohol consumption per week, BMI, cUHDRS and time of follow-up).

Associations between modifiable and non-modifiable factors were established in two steps:

1. Due to the participant-clustered structure of data (repeated measurements per individual) a mixed-effect random forest (MERF) was calibrated. The MERF is a supervised machine learning model with random effects parameters, added to fixed effects drawn from a random forest model. This method makes it possible to take into account multiple sources of random variability (intra- and inter-individual) and to draw robust statistical conclusions about variables with possible interaction^1^.
2. For model calibration, individuals were included as random effects at intercept and slope with age as the timescale. Age at interview, CAG repeats, sociodemographic factors, health behaviors, and health status variables were included as fixed effects. Data were divided into training and validation sets (75 and 25% respectively). The evaluation and optimization of the MERF model parameters were developed using an expectation-maximization algorithm with Generalized Log Likelihood (GLL) as a measure of training loss. The random forest did not include a maximum of features, depths, nodes, or samples for fixed effects. The minimum number of leaves was 1, and of samples by leaf was 2. The number of estimators was 300.
3. The explanation of the MERF model results was carried out using the Shapley additive explanation method (SHAP), based on game theory concepts^2^. The SHAP method decomposes the output of a machine learning model by the sum of the impacts of each variable to calculate a SHAP value. Each SHAP value represents the contribution of each variable to each prediction estimated by the model. With the absolute mean of SHAP values [mean(|shap|)], the association and importance of each variable may be estimated and studied. SHAP also allows the manipulation of models with complex behaviors involving multiple interactions between variables. To illustrate the results, plots of the predictions in terms of their importance assigned to the model and SHAP method were used, as well as plots of the partial dependence and the interactions with trend lines divided by categories and by the median for continuous variables to facilitate the interpretation.

Several sensitivity analyses were performed to assess the robustness of the results. First, a similar MERF model was calibrated with the CAG-Age Product (CAP)^3^ to reduce the impact of age, CAG number repeats, and their interaction on the results. A linear mixed model was fitted with the variables and interactions suggested by the MERF to assess possible model biases. The analysis was repeated for each component of the cUHDRS (TFC, TMS, Stroop W, and SDMT) separately to account for potential bias generated by any of the assessments. The analysis was performed with the 3,708 participants with at least one cUHDRS measure to evaluate possible selection bias. To evaluate potential selection biases in the analysis population, we conducted the primary analyses again using an HD-ISS criteria-based stage 0-1 population (including individuals with CAG≥40 and using baseline values of TMS, SDMT adjusted for education levels)^4^.To prevent associations from being driven by the number of participants in the exposure set and the main outcome, and to assess the impact of under-representation of exposure to certain variables such as heavy alcohol use, drug use, or obesity, we added propensity score weighting^5^ to the calibrated linear mixed model calibrated to the variables suggested by MERF.

Supplementary Results

*Example of interpretation*

Figure 2, right-hand panel, shows the SHAP values for each of the individuals, represented by single dots, per variable and as a function of variable values. For example, low ages in blue have negative SHAP values and affect negatively the cUHDRS score. For each presymptomatic mutation carrier, it was possible to add up the SHAP values corresponding to him or her and explain the distance of his or her cUHDRS score from the population mean. Figure S3 shows examples. For participant 1, his educational level of 6 adds 0.37 (SHAP value) to the overall mean (17.01+0.37=17.38), the 41 CAG repetitions add 0.37 (17.38+0.37=17.75), his smoking decreases his predicted cUHDRS score by 0.32 (17.75-0.32=17.43) and so on to reach a predicted cUHDRS score of 18.02. By estimating the absolute value of the mean of the SHAP values [mean(|shap|)] for each variable, we were able to determine their level of contribution to the model when estimating the predictions (Figure 2, left-hand panel).

*Sensitivity Analysis results*

Replicating the analyses with CAP^3^ replacing age and number of CAG gave results similar to the main findings, with an MAE of 0.24 and an MSE of 0.10 (Figure S6). Calibration of a linear mixed model with individual-level slope and intercept random effects led to significant results for each variable identified by the MERF and an MAE of 0.53 (Table S2). Separate analysis of each measure making up the cUHDRS also showed results comparable to those of the main analysis, with no influence of one measure on the others (Figure S7). The analysis performed on patients with a single cUHDRS measurement also produced results similar to the main findings (Figure S8). Replication of the analyses with the 0-1 status population according to the HD-ISS criteria (n=2471) showed similar results to the main results (Figure S9). Finally, most of the findings of our study have been preserved following analysis using the linear mixed model with propensity score weighting^5^ (Table S3). In this model, age, GAC, BMI, educational level, pharmacological treatment of depression, tobacco use, and sex retained their association with the cUHDRS trajectory.

References

1. Hajjem A, Bellavance F, Larocque D. Mixed-effects random forest for clustered data. *Journal of Statistical Computation and Simulation* 2014; **84**(6): 1313-28.

2. Lundberg SM, Erion GG, Lee S-I. Consistent individualized feature attribution for tree ensembles. *arXiv preprint arXiv:180203888* 2018.

3. Warner JH, Long JD, Mills JA, et al. Standardizing the CAP Score in Huntington's Disease by Predicting Age-at-Onset. *J Huntingtons Dis* 2022; **11**(2): 153-71.

4. Tabrizi SJ, Schobel S, Gantman EC, et al. A biological classification of Huntington's disease: the Integrated Staging System. *Lancet Neurol* 2022; **21**(7): 632-44.

5. Schuler MS, Chu W, Coffman D. Propensity score weighting for a continuous exposure with multilevel data. *Health Serv Outcomes Res Methodol* 2016; **16**(4): 271-92.


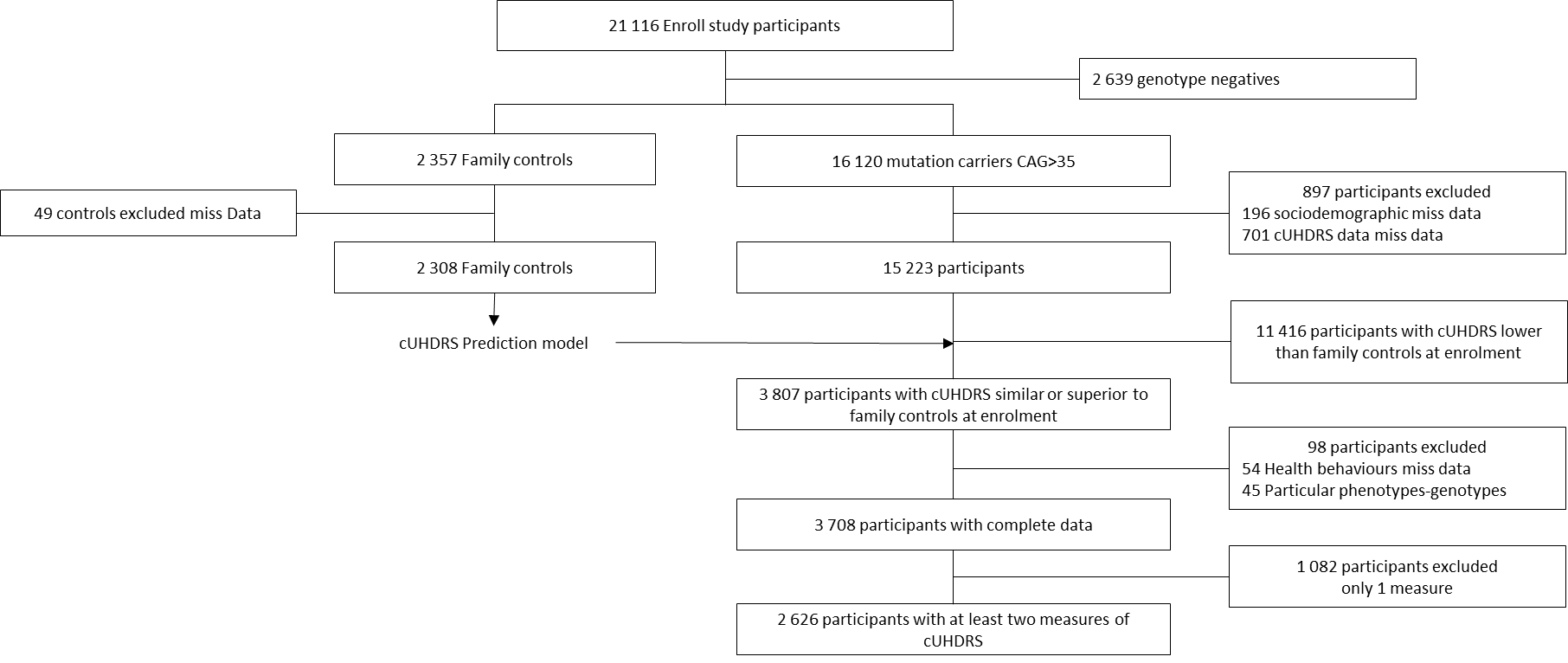
**Figure S1. Analysis population selection flowchart extended**

**Figure S2. Mixed effect random forest fully adjusted, Panel A: Association between the cUHDRS score predicted and cUHDRS score observed in the calibration sample. Panel B: Distribution of betas by subject associated with the random effect adjusted.**

**
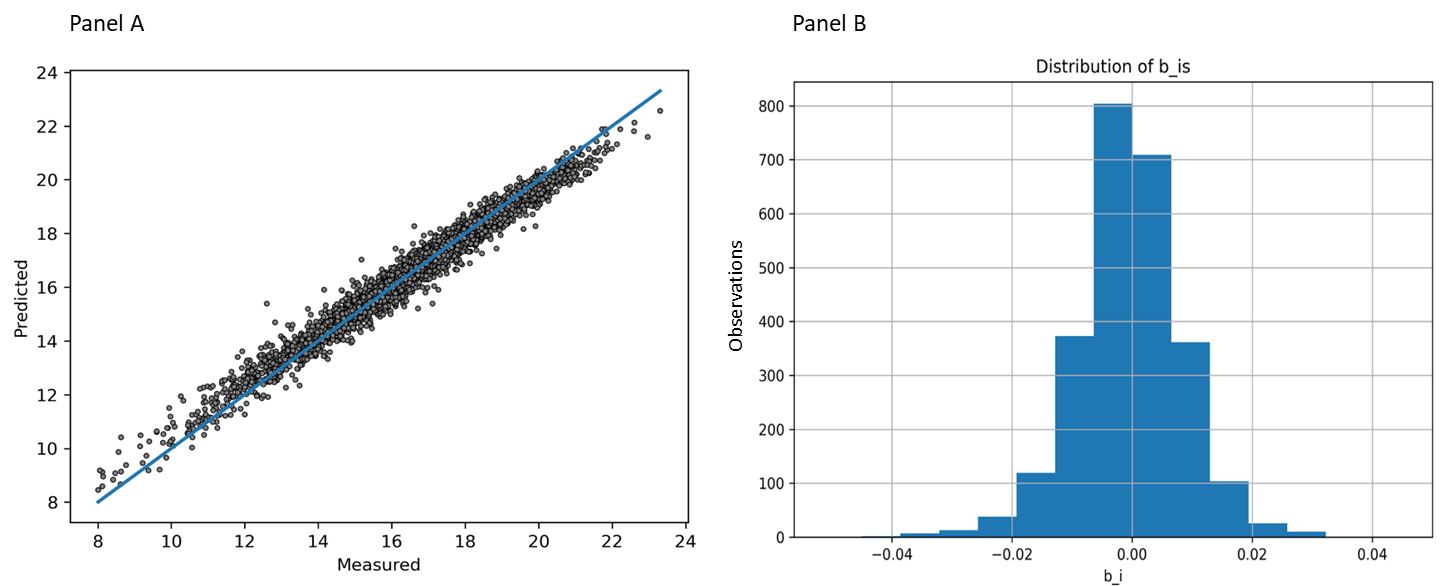
**

*Predictions were drawn from a mixed-effect Random Forest with a training sample adjusted for sociodemographic variables, health behaviors, comorbidities, depression and anxiety treatments and the number of CAG repeats.*

*
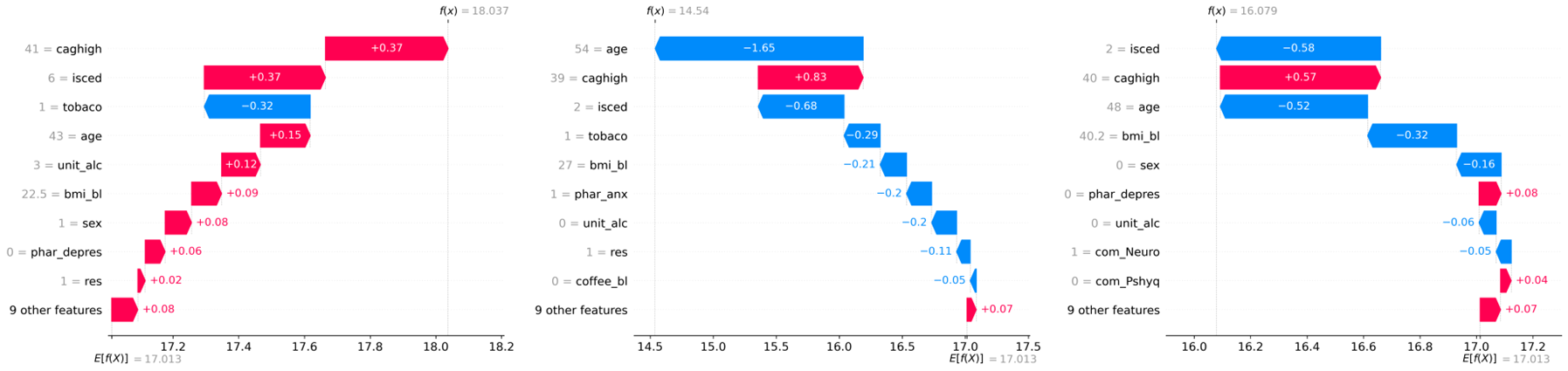
***Figure S3. Waterfall plots to individual prediction explanation**

*SHAP values by participants of the predictions of fully adjusted mixed effect random forest model. Red: increases the prediction of the cUHDRS with respect to the global mean. Blue: decreases the prediction of the cUHDRS with respect to the global mean. Ranges of feature value: age [18-80], number of CAG repeats [36-55], Education level [0=low, 1=medium, 2=high], BMI [18-36], Pharmacological depression and anxiety treatment [0=Non-1=Yes], units of alcohol per week [0-30], tobacco and drugs consumption [0=Non-1=Yes], sex [0=man-1=women], all comorbidities history [0=Non-1=Yes], marital status [0=single, 1=in couple], Residence in a city [0=Non-1=Yes].*

**
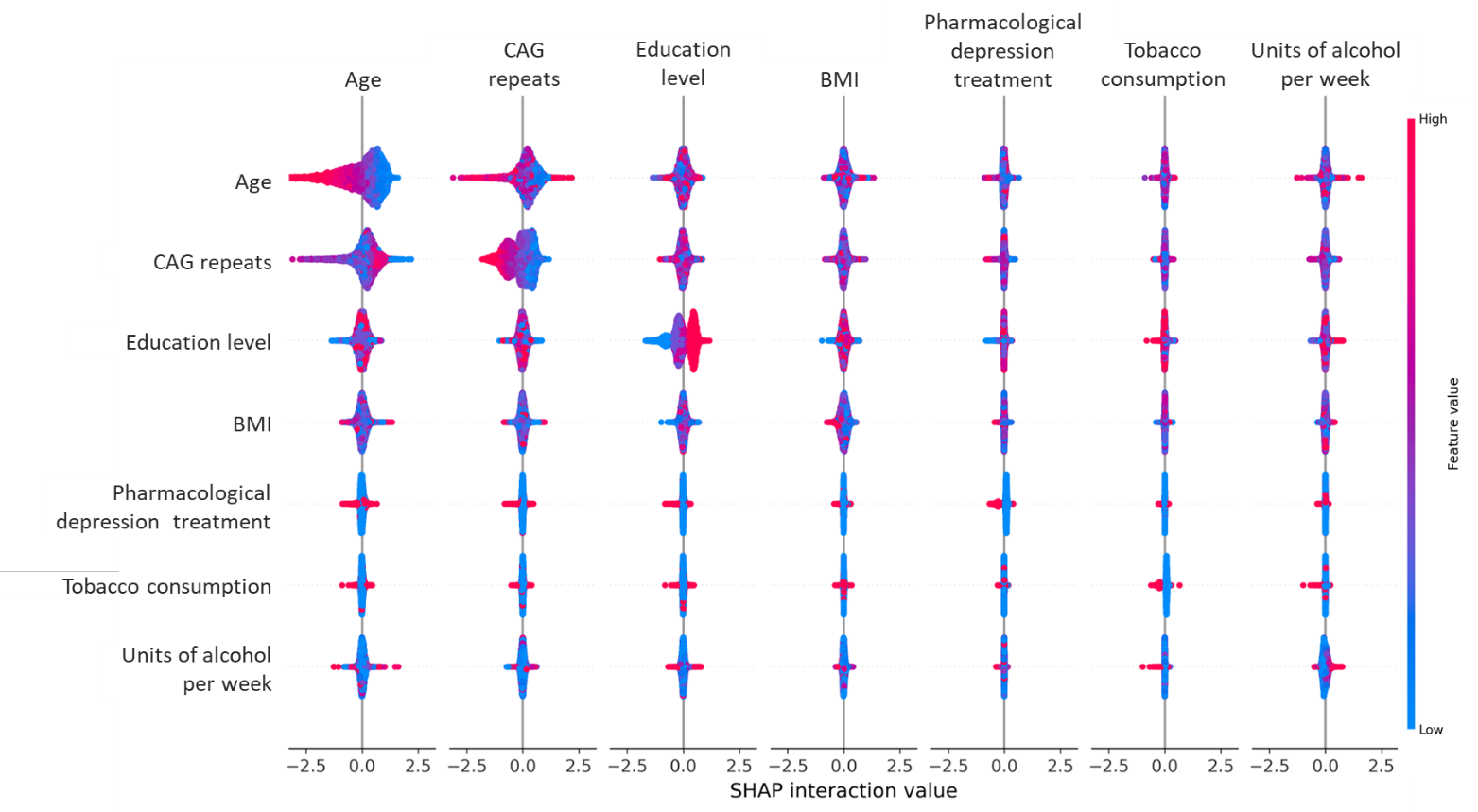
Figure S4. Summary plot of SHAP values of interactions between most relevant variables**

*SHAP values of the predictions of fully adjusted mixed effect random forest model. Ranges of feature value: age [18-80], number of CAG repeats [36-55], Education level [0-6 ISCED levels], BMI [18-36], Pharmacological depression and anxiety treatment [0=Non-1=Yes], units of alcohol per week [0-30], tobacco and drugs consumption [0=Non-1=Yes], sex [0=man-1=women], all comorbidities history [0=Non-1=Yes], marital status [0=single, 1=in couple], Residence in a city [0=Non-1=Yes].*

**Figure S5. SHAP values of the interactions between the most relevant HD presymptomatic factors in**
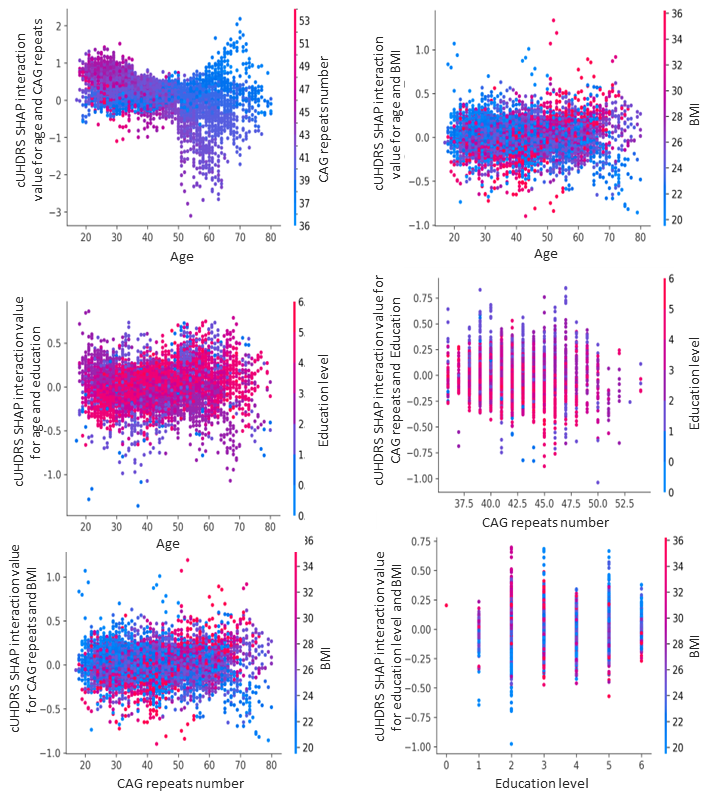
**the prediction of cUHDRS with a mixed-effect random forest model.**

*SHAP interaction values drawn of the predictions of fully adjusted mixed effect random forest model*.

**
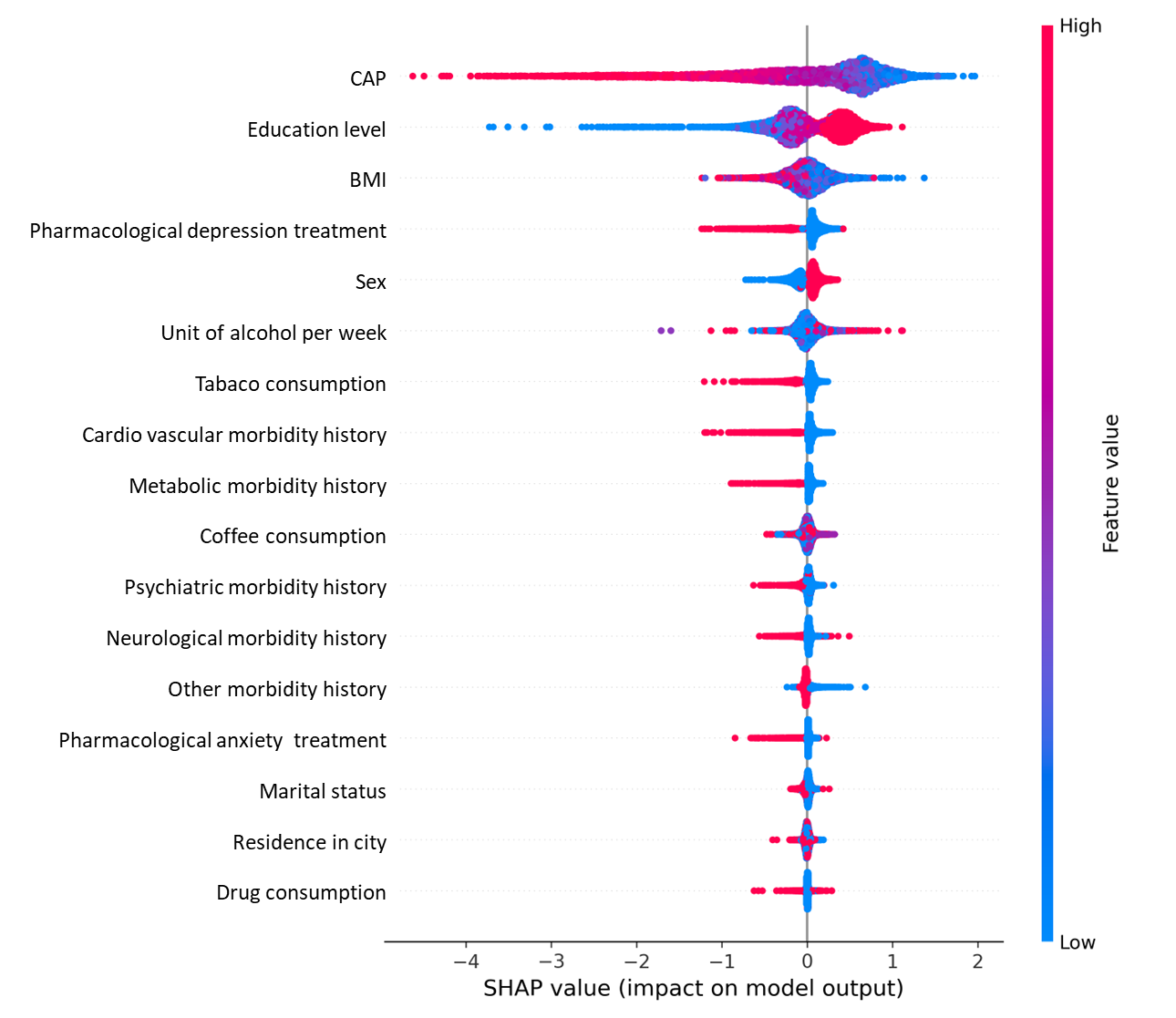
Figure S6. Summary plot of the SHAP values for each prediction of cUHDRS including CAP organized by level of relevance of each variable**

*SHAP values of the predictions of fully adjusted mixed effect random forest model. Ranges of feature value: age [18-80], number of CAG repeats [36-55], Education level [0=low, 1=medium, 2=high], BMI [18-36], Pharmacological depression and anxiety treatment [0=Non-1=Yes], units of alcohol per week [0-30], tobacco and drugs consumption [0=Non-1=Yes], coffee consumption (0=non, 1=currently, 2=more 3 cups per day),* *sex [0=man-1=women], all comorbidities history [0=Non-1=Yes], marital status [0=single, 1=in couple], Residence in a city [0=Non-1=Yes].*

**
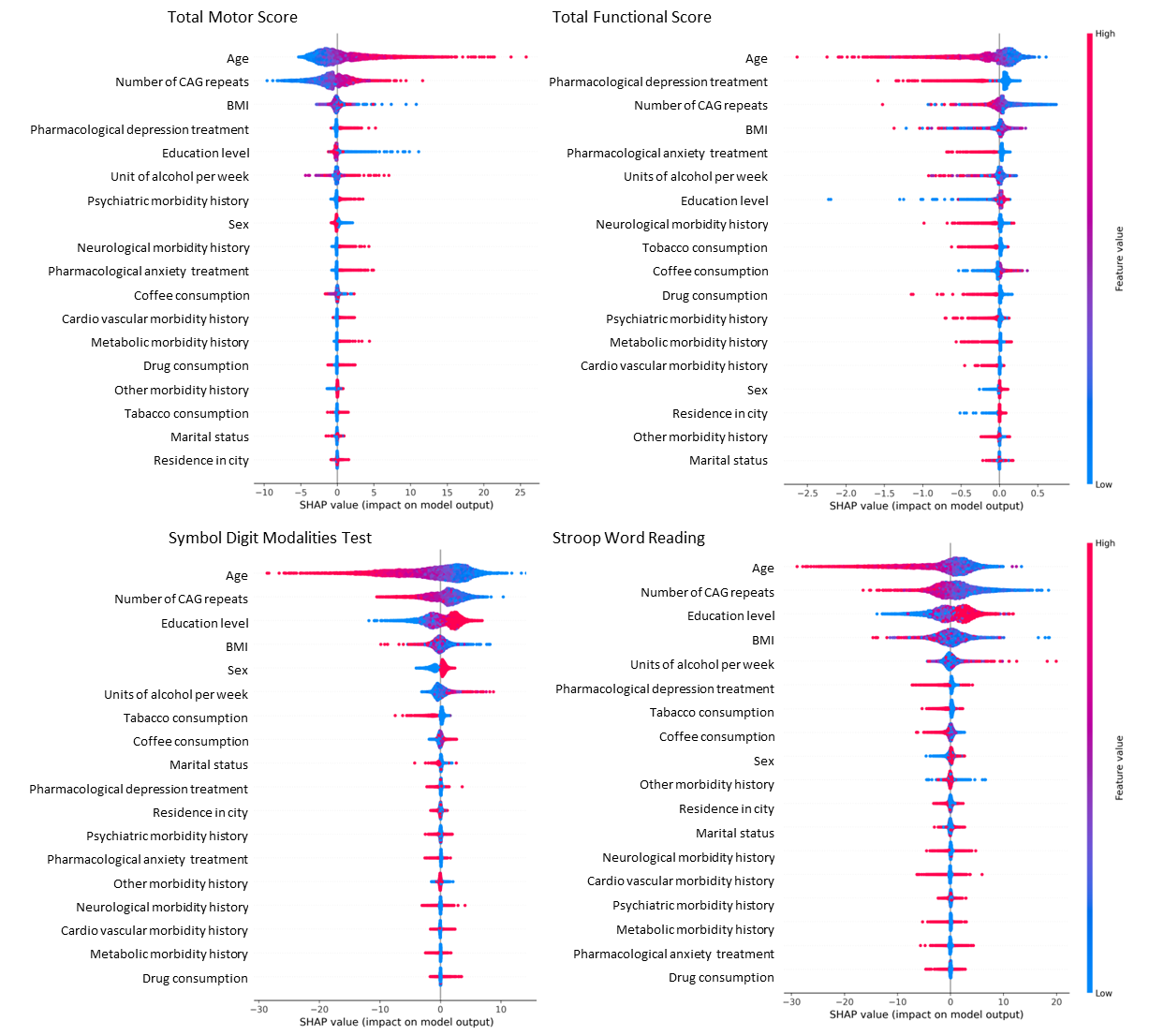
Figure S7. Summary plot of the SHAP values for each prediction of cUHDRS by each total motor score, total functional score, symbol digit modalities test and Stroop word reading.**

*SHAP values of the predictions of fully adjusted mixed effect random forest model. Ranges of feature value: age [18-80], number of CAG repeats [36-55], Education level [0=low, 1=medium, 2=high], BMI [18-36], Pharmacological depression and anxiety treatment [0=Non-1=Yes], units of alcohol per week [0-30], tobacco and drugs consumption [0=Non-1=Yes], coffee consumption (0=non, 1=currently, 2=more 3 cups per day), sex [0=man-1=women], all comorbidities history [0=Non-1=Yes], marital status [0=single, 1=in couple], Residence in a city [0=Non-1=Yes].*

**Figure S8. Summary plot of the SHAP values for each prediction of cUHDRS including participants with only one measure organized by the level of relevance of each variable**

*
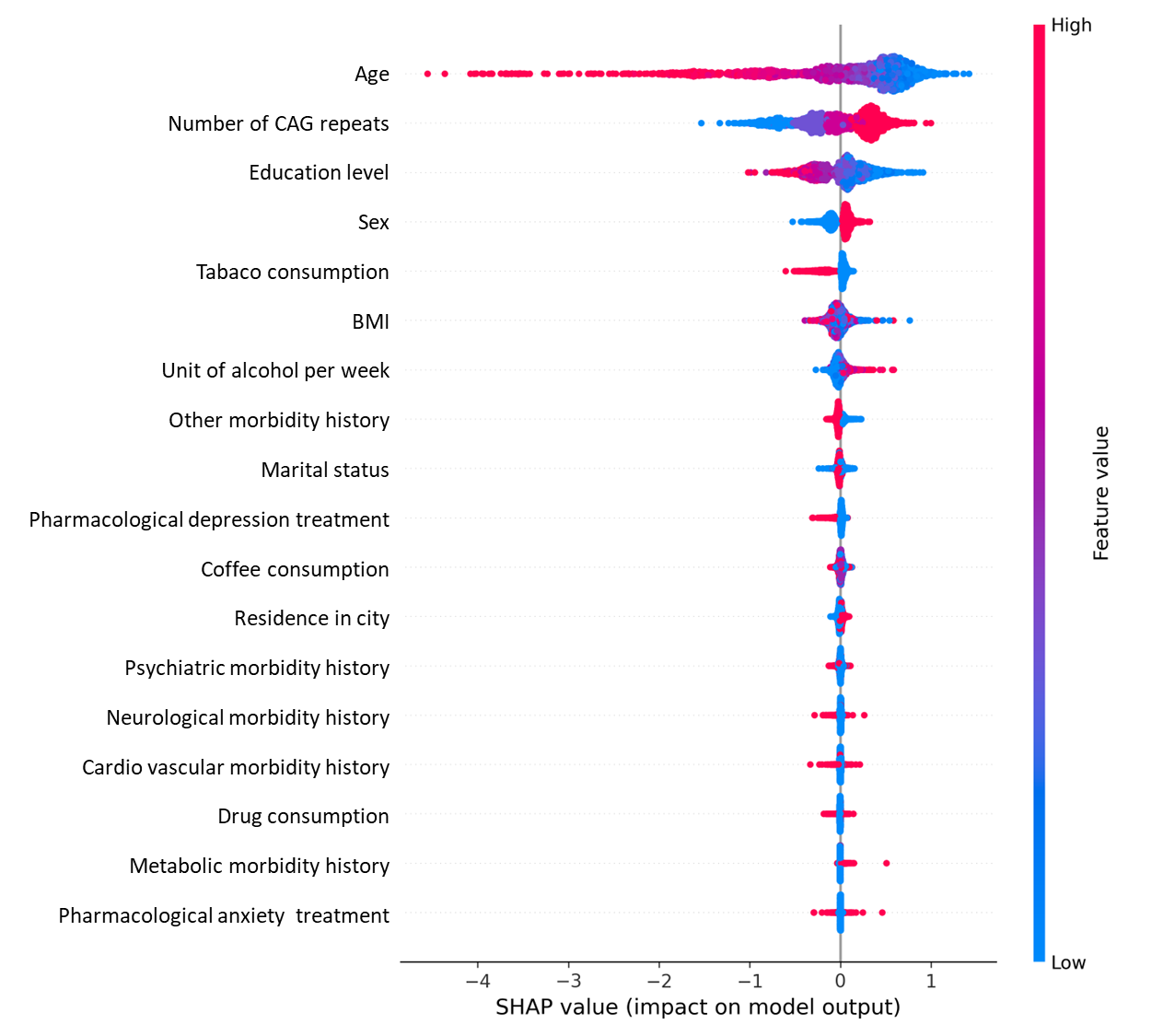
*

*SHAP values of the predictions of fully adjusted mixed effect random forest model. Ranges of feature value: age [18-80], number of CAG repeats [36-55], Education level [0=low, 1=medium, 2=high], BMI [18-36], Pharmacological depression and anxiety treatment [0=Non-1=Yes], units of alcohol per week [0-30], tobacco and drugs consumption [0=Non-1=Yes], sex [0=man-1=women], all comorbidities history [0=Non-1=Yes], marital status [0=single, 1=in couple], Residence in a city [0=Non-1=Yes].*

**Figure S9. Summary plot of the SHAP values for each prediction of cUHDRS calibrated with particpantns in stage 0-1 in HD-ISS organized by level of relevance of each variable**

**
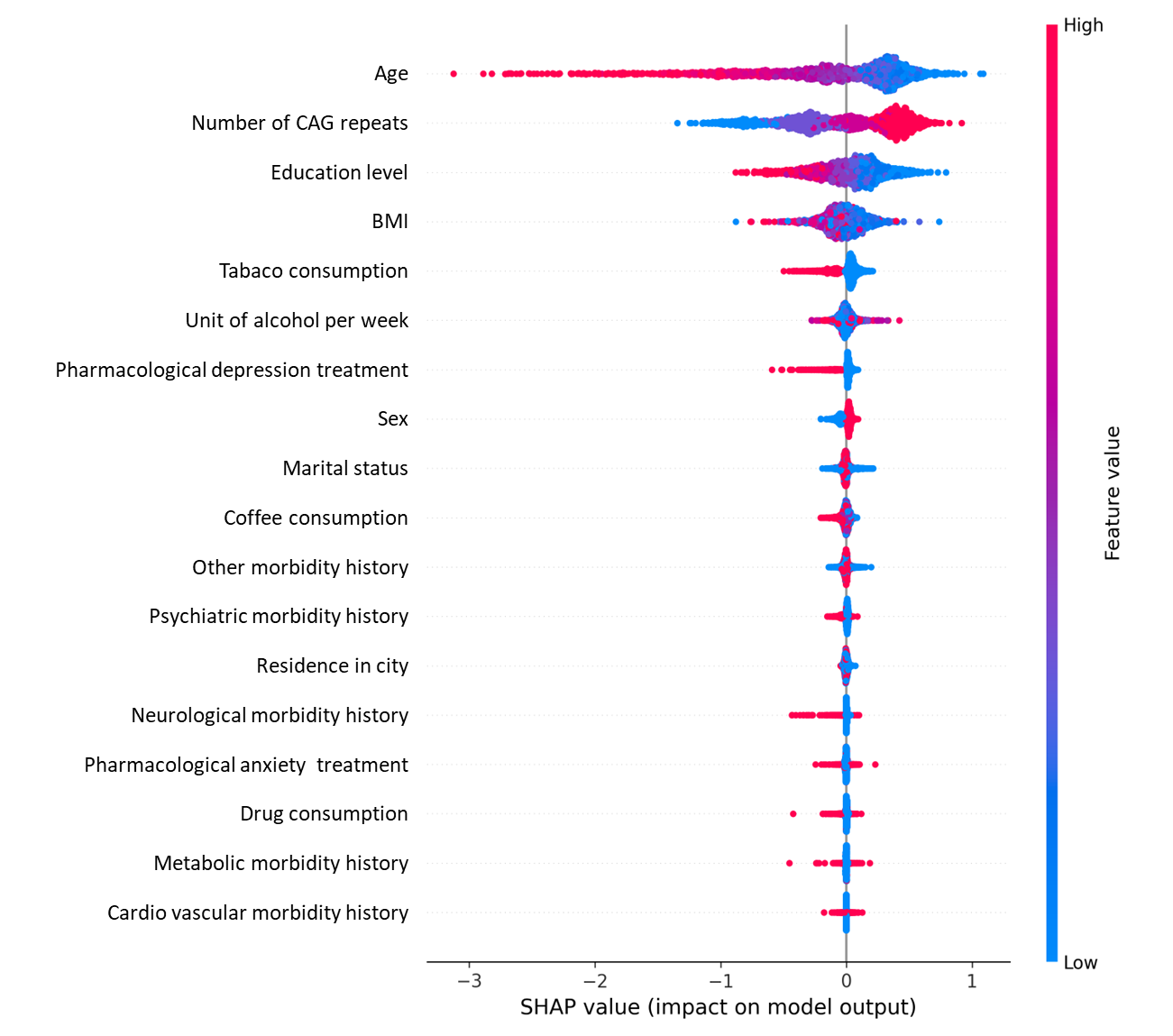
**

*SHAP values of the predictions of fully adjusted mixed effect random forest model. Ranges of feature value: age [18-80], number of CAG repeats [36-55], Education level [0=low, 1=medium, 2=high], BMI [18-36], Pharmacological depression and anxiety treatment [0=Non-1=Yes], units of alcohol per week [0-30], tobacco and drugs consumption [0=Non-1=Yes], sex [0=man-1=women], all comorbidities history [0=Non-1=Yes], marital status [0=single, 1=in couple], Residence in a city [0=Non-1=Yes].*

**Table S1. Comparison between those with at least two measures of cUHDRS included in the study and those with only one measure of cUHDRS.**

| **Characteristics of participants** | **Included (N=2626)** | **Excluded (N=1082)** | **p-value** |
| --- | --- | --- | --- |
| Sex |  |  |  |
| Women | 1510 (57.5%) | 644 (59.5%) | 0.258 |
| Man | 1116 (42.5%) | 438 (40.5%) |  |
| Age, mean (SD) | 40.16 (12.60) | 38.91 (12.63) | 0.006 |
| CAG repeats, mean (SD) | 41.97 (2.52) | 42.16 (2.62) | 0.037 |
| Education level |  |  |  |
| Low | 102 (9.4%) | 330 (12.6%) | 0.007 |
| Medium | 548 (50.6%) | 1216 (46.3%) |  |
| High | 432 (39.9%) | 1080 (41.1%) |  |
| Marital status |  |  |  |
| Single | 1749 (66.6%) | 718 (66.4%) | 0.886 |
| In couple | 877 (33.4%) | 364 (33.6%) |  |
| Residence in City |  |  |  |
| Non | 1429 (54.4%) | 574 (53.0%) | 0.448 |
| Yes | 1197 (45.6%) | 508 (47.0%) |  |
| Alcohol consumption (units), mean (SD) | 4.08 (7.23) | 3.60 (6.13) | 0.056 |
| Tobacco consumption |  |  |  |
| Non | 2010 (76.5%) | 786 (72.6%) | 0.012 |
| Yes | 616 (23.5%) | 296 (27.4%) |  |
| Coffee consumption |  |  |  |
| Non | 976 (37.2%) | 180 (16.6%) | < 0.001 |
| Currently | 969 (36.9%) | 527 (48.7%) |  |
| More than 3 cups per day | 681 (25.9%) | 375 (34.7%) |  |
| Abused drugs |  |  |  |
| Non | 2243 (85.4%) | 863 (79.8%) | < 0.001 |
| Yes | 383 (14.6%) | 219 (20.2%) |  |
| BMI (Kg/m^2^) | 26.01 (5.36) | 26.14 (5.53) | 0.529 |
| Comorbidities |  |  |  |
| Cardio-Vascular | 349 (13.3%) | 110 (10.2%) | 0.009 |
| Metabolic | 331 (12.6%) | 103 (9.5%) | 0.008 |
| Neurologic | 393 (15.0%) | 171 (15.8%) | 0.518 |
| Psychiatric | 710 (27.0%) | 255 (23.6%) | 0.029 |
| Others | 1836 (69.9%) | 650 (60.1%) | < 0.001 |
| Pharmacological treatment for |  |  |  |
| Anxiety | 182 (6.9%) | 73 (6.7%) | 0.841 |
| Depression | 402 (15.3%) | 148 (13.7%) | 0.204 |
| cUHDRS, mean (SD) | 17.16 (1.38) | 16.93 (1.51) | < 0.001 |

**Table S2. The linear mixed model fitted based on the relevant Mixed effect random forest factors.**

|  | **cUHDRS** | | |
| --- | --- | --- | --- |
| ***Variables*** | ***Estimates*** | ***CI*** | ***p*** |
| (Intercept) | -25.21 | -34.66 – -15.76 | **<0.001** |
| Age | 13.67 | 12.17 – 15.17 | **<0.001** |
| Age^2 | 1.02 | 0.28 – 1.77 | **0.007** |
| CAG repeats | 2.32 | 1.87 – 2.76 | **<0.001** |
| CAG repeats^2 | -0.03 | -0.04 – -0.03 | **<0.001** |
| BMI | -0.02 | -0.03 – -0.01 | **<0.001** |
| Education level | 0.24 | 0.20 – 0.28 | **<0.001** |
| Pharmacological depression treatment | -0.53 | -0.62 – -0.44 | **<0.001** |
| Alcohol consumption (units per week) | 0.01 | 0.01 – 0.02 | **0.001** |
| Tobacco consumption | -0.44 | -0.55 – -0.32 | **<0.001** |
| Coffee consumption | 0.1 | -0.00 – 0.20 | 0.051 |
| Sex | 0.32 | 0.22 – 0.42 | **<0.001** |
| Age*CAG-repeats | -0.36 | -0.40 – -0.33 | **<0.001** |
| Age^2*CAG-repeats | -0.04 | -0.06 – -0.02 | **<0.001** |
| BMI*Age | 0 | -0.01 – 0.01 | 0.611 |
| Education-level*Age | 0.04 | 0.00 – 0.08 | **0.04** |
| **Random Effects** | | | |
| σ^2^ | 0.68 | | |
| τ_00_ _subject_ | 1.20 | | |
| τ_11_ _subject. age2_ | 0.40 | | |
| ρ_01_ _subject_ | 0.50 | | |
| ICC | 0.70 | | |
| N _subject_ | 2626 | | |
| Observations | 9886 | | |
| Marginal R^2^ / Conditional R^2^ | 0.435 / 0.833 | | |

**Table S3. The linear mixed model with propensity score weighting fitted based on the relevant Mixed effect random forest factors.**

|  | **cUHDRS** | | |
| --- | --- | --- | --- |
| *Predictors* | *Estimates* | *CI* | *p* |
| (Intercept) | -54.32 | -71.41 – -37.23 | **<0.001** |
| Age | 16.81 | 14.08 – 19.54 | **<0.001** |
| Age^2 | -0.55 | -0.66 – -0.45 | **<0.001** |
| CAG repeats | 3.74 | 2.93 – 4.55 | **<0.001** |
| CAG repeats^2 | -0.05 | -0.06 – -0.04 | **<0.001** |
| BMI | -0.02 | -0.04 – -0.00 | **0.041** |
| Education level | 0.17 | 0.10 – 0.23 | **<0.001** |
| Pharmacological depression treatment | -0.35 | -0.49 – -0.21 | **<0.001** |
| Alcohol consumption (units per week) | 0.01 | -0.00 – 0.03 | 0.159 |
| Tobacco consumption | -0.34 | -0.54 – -0.14 | **0.001** |
| Coffee consumption | 0.1 | -0.07 – 0.27 | 0.246 |
| Sex | 0.22 | 0.06 – 0.39 | **0.009** |
| Age*CAG-repeats | -0.44 | -0.50 – -0.38 | **<0.001** |
| Age^2*CAG-repeats | -0.01 | -0.03 – 0.02 | 0.572 |
| BMI*Age | 0.1 | 0.02 – 0.17 | **0.011** |
| Education-level*Age | 0.09 | 0.02 – 0.17 | **0.014** |
| **Random Effects** | | | |
| σ^2^ | 0.64 | | |
| τ_00_ _subjid_ | 2.57 | | |
| τ_11_ _subjid.age2_ | 4.62 | | |
| ρ_01_ _subjid_ | 0.45 | | |
| ICC | 0.92 | | |
| N _subjid_ | 2626 | | |
| Observations | 9886 | | |
| Marginal R^2^ / Conditional R^2^ | 0.164 / 0.932 | | |
